# Poster Session II - A199 EVALUATION OF PERIPHERAL ABSOLUTE EOSINOPHIL COUNT AS A BIOMARKER OF DISEASE ACTIVITY IN EOSINOPHILIC ESOPHAGITIS

**DOI:** 10.1093/jcag/gwaf042.199

**Published:** 2026-02-13

**Authors:** K Chan, A Wilson

**Affiliations:** Western University, London, ON, Canada; Medicine, Western University, London, ON, Canada

## Abstract

**Background:**

At present, the gold standard for eosinophilic esophagitis (EoE) diagnosis and monitoring is upper gastrointestinal endoscopy (EGD) and biopsy of the esophageal mucosa. Unfortunately, the need for multiple and/or sequential treatments in this population can lead to patients needing recurrent, invasive EGDs for evaluation of disease activity and treatment response over time.

**Aims:**

To evaluate the association between peripheral absolute eosinophil count (pAEC) and esophageal eosinophilia as a tool for monitoring EoE disease activity without the need for EGD.

**Methods:**

A cross-sectional study is ongoing in adult EoE patients seen at a tertiary care centre in London, Canada. Participants who underwent an EGD with esophageal biopsy and had an available pAEC are included. Demographic data, pAEC, histological disease activity, and endoscopic findings were collected from each EGD encounter. Participants were evaluated for the association between 1) histological disease remission (<15 eosinophils per high powered field averaged between mid and distal esophageal biopsies), 2) individual EGD findings and pAEC respectively. The relationship between pAEC and mean esophageal eosinophilia was assessed using correlation analyses. A receiver operator characteristic (ROC) analysis was used to identify a threshold pAEC associated with histological disease activity or EGD findings, where significant.

**Results:**

To date, 94 participants with 142 EGD encounters are included in the preliminary analyses. The median pAEC was higher in participants with active EoE on histology (0.3 x10^9^ cells/L, interquartile range, IQR=0.6x10^9^ cells/L) versus those in remission (0.2 x10^9^ cells/L, interquartile range, IQR=0.6x10^9^ cells/L, p = 0.0095). The median pAEC was higher in participants with white exudate on EGD (0.4 x10^9^ cells/L, interquartile range, IQR=0.3x10^9^ cells/L) versus those without (0.3 x10^9^ cells/L, interquartile range, IQR=0.3x10^9^ cells/L, p = 0.01). Additionally, the mean esophageal eosinophil count was positively correlated with pAEC (r = 0.24, p = 0.0026). A threshold pAEC=0.15 x 10^9^ cells/L separated those with histological disease remission from those without (area under the curve, AUC=0.63, 95% confidence interval, CI = 0.53-0.74, p = 0.015). A threshold pAEC=0.37 x 10^9^ cells/L separated those with white exudate on EGD from those without (AUC=0.73, 95%CI=0.59-0.87, p = 0.013).

**Conclusions:**

To date, pAEC is positively correlated with histological disease activity in EoE, with higher pAEC associated with the presence of esophageal eosinophilia. Additionally, we see white exudate on EGD is associated with higher pAEC. With completion of this study, we hope to determine if pAEC is a simple and clinically-actionable biomarker of EoE disease activity. This may reduce the need for repeated, invasive endoscopies in an otherwise healthy population.

**Funding Agencies:**

None

